# Evidence informed health policy making: role of evidence in six health policies in India and Nigeria

**DOI:** 10.1186/1472-6963-14-S2-P26

**Published:** 2014-07-07

**Authors:** Mahua Das, Bassey Ebenso, Reinhard Huss, Bindiya Rawat, Nkoli Guru, Guliano Russo, Yme Van Den Berg, Weerasak Puttasari, Tolib Mirzoev

**Affiliations:** 1 Nuffield Centre for International Health and Development, University of Leeds, West Yorkshire, UK; 2 Association for Stimulating Know How, New Delhi, India; 3 College of Medicine, University of Nigeria Enugu Campus, Enugu, Nigeria; 4 Instituto De Higiene E Medicina Tropical, Lisbon, Portugal; 5 Het Koninklijk Instituut Voor De Tropen, Netherlands, The Netherlands; 6 Mahidol University, Bangkok, Thailand

## Introduction

The importance of evidence-informed health policy making is widely recognised and frameworks exist to help understand the roles of different types of evidence [1,2]. However, there is limited application of these frameworks in empirical studies, particularly to inform learning and improve evidence-informed health policy development in LMICs. The objective of this presentation is to discuss results of comparative analysis of the role of evidence in health policy development in six case studies from India and Nigeria, and share the resultant lessons.

## Methods

A conceptual framework linking policy and evidence processes drew on literature and guided the study. Qualitative data was collected using documents review and in-depth interviews and analysed using framework approach. We analysed the role of evidence in three types of policies in each country: internationally-’prominent’ (e.g. AIDS control); internationally-’neglected’ (e.g. oral health) and health systems policy (e.g. human resources).

## Results

Overall, policy development in all six policies was perceived to be evidence-informed. Both formal (e.g. research articles and HMIS) and informal (e.g. stakeholder experiences) evidence were used in India. In Nigeria, reliance was mostly on formal evidence. Although a range of types and sources of evidence (e.g. national surveys, policy document) influenced different stages of policy process across case studies, evidence was largely used to establish the need for a policy as ‘evidence for policy’ and less for analysing policy options as ‘evidence on policy’. The role of evidence was influenced by factors within policy actors and policy making organisations and their context. Such factors included the perceived characteristics of robust evidence, its availability and accessibility, national and international political commitments; and different policy and evidence processes.

## Conclusion

The role of evidence in health policy development is interpreted as a relationship between policy and evidence processes, and informed by the influence of policy actors and their national and international context. The potential impact of this research includes recommendations for strengthening evidence-informed and context-specific policymaking. Our framework and findings improve our understanding of the role of evidence in health policy development and how to promote evidence-informed policy policy processes in different contexts.
